# Supplementary material for: IterCluster: a barcode clustering algorithm for long fragment read analysis
Source: PeerJ. 2020 Mar 24;8:e8431. doi: 10.7717/peerj.8431 (PMC7100596; doi:10.7717/peerj.8431)
Supplement: Supplemental Information 1 [file peerj-08-8431-s001.docx]

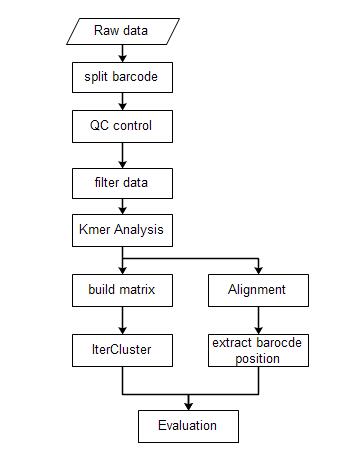


The workflow of IterCluster and its evaluation pipeline

**The script used in each step:**

|  | stLFR data | 10x data |
| --- | --- | --- |
| Split barcode | 01.split_barcode_stlfr.pl | Longranger, 01.split_10X_reads_new.pl |
| QC filter dup & adapter | 02.SOAPfilter_stlfr.sh | 02.SOAPfilter_10x.sh |
| Filter N and small barcode | 03.Reads_filter.pl | 03.Reads_filter.pl |
| Kmer Analysis | 04.kmer.sh | 04.kmer.sh |
| Alignment | 05.aln_run.sh | 05.aln_run.sh |
| Get barcode position | 06.get_barcode_position.pl | 06.get_barcode_position.pl |
| Build matrix | get_matrix_run.sh | get_matrix_run.sh |
| IterCluster | IterCluster-MCL.sh | IterCluster-MCL.sh |
| Evaluation | 07.Single_MclIterCuster_evaluate.pl | 07.Single_MclIterCuster_evaluate.pl |

More detail can be found on IterCluster’s github page.

**The adapter used in SOAPfilter:**

The adapter1 is CTGTCTCTTATACACATCTTAGGAAGACAAGCACTGACGACATGA.

The adapter2 is TCTGCTGAGTCGAGAACGTCTCTGTGAGCCAAGGAGTTGCTCTGG.
